# Novel sequence variants in the *TLR6* gene associated with advanced breast cancer risk in the Saudi Arabian population

**DOI:** 10.1371/journal.pone.0203376

**Published:** 2018-11-02

**Authors:** Abdelhabib Semlali, Mikhlid Almutairi, Mahmoud Rouabhia, Narasimha Reddy Parine, Abdullah Al Amri, Nouf S. Al-Numair, Yousef M. Hawsawi, Mohammad Saud Alanazi

**Affiliations:** 1 Groupe de Recherche en Écologie Buccale, Département de stomatologie, Faculté de Médecine Dentaire, Université Laval, Québec, Qc, Canada; 2 Genome Research Chair, Department of Biochemistry, College of Science, King Saud University, Riyadh, Kingdom of Saudi Arabia; 3 Zoology Department, College of Science, King Saud University, Riyadh, Kingdom of Saudi Arabia; 4 Department of Genetics, Research Center, King Faisal Specialist Hospital and Research Center, Riyadh, Kingdom of Saudi Arabia; 5 College of Medicine, Alfaisal University, Riyadh, Saudi Arabia; University of Cambridge, UNITED KINGDOM

## Abstract

Herein, we evaluated the association of the Toll-like receptor 6 (*TLR6*) single nucleotide polymorphisms (SNPs) rs3796508 (Val327Met) and rs5743810 (Ser249Pro) with breast cancer (BC) susceptibility in Saudi Arabian women, using in silico analysis. We found no significant differences in genotypic and allelic frequencies for rs3796508 between the BC patients (*n* = 127) and healthy individuals (*n* = 116). However, 86% of the BC patients, versus 98% of the healthy controls, carried the rs5743810 Pro allele (OR = 0.103, CI = 0.036–0.293, *P* = 0.00001). Advanced analysis based on the comparison of the estrogen receptor (ER)-positive and -negative patients with the healthy controls indicated a significant association between rs5743810 allelic frequency and BC risk protection (OR = 0.100, CI = 0.034–0.297, *P* = 0.00001 for ER^+^ BC cases; OR = 0.102, CI = 0.033–0.318, *P* = 0.00001 for ER^−^BC cases). Furthermore, rs5743810 was associated with BC risk protection at either above or below 48 years of age at diagnosis (OR = 0.101, CI = 0.022–0.455, *P* = 0.00037 for age ≤48 years; OR = 0.120, CI = 0.028–0.519, *P* = 0.00087 for age >48 years). Such associations were not found for rs3796508. In silico analysis indicated that these SNPs had neutral effects within the TLR6 structure, confirming the protective role of rs5743810. Our findings therefore suggest a strong association between rs5743810 and protection against BC risk in Saudi Arabian women. Importantly, the rs5743810 Pro allele could be a potential BC diagnostic biomarker in this ethnic population.

## Introduction

In recent years, breast cancer (BC) has become the most frequently diagnosed cancer among women worldwide, with approximately 2.5 million cases in 2015 [1]. The lethal disease is also one of the most prevalent cancers among women living in Gulf Cooperation Council countries, including Saudi Arabia [2]. In Saudi Arabia, BC is considered the leading cause of female mortality among all other cancers [3], being more prevalent among young women in comparison to women suffering from BC in Western countries [4]. According to the 2013 cancer incidence report from the Saudi Health Council, BC cases accounted for approximately 16% of all malignances among adults of both sexes and approximately 29% of all malignances among Saudi Arabian females.

The relationship between the dysregulation of innate immunity-related genes and BC development is widely documented in the literature, including that of Toll-like receptors (TLRs)[5–7]. Importantly, the human TLRs are essential components of the innate immunity that provides host protection against infections caused by both viral and bacterial organisms[5]. TLR expression is induced by different types of immune cells, such as dendritic cells and macrophages [8]; however, their expression is also found in non-immune system cells, such as oral mucosal cells and skin keratinocytes [9]. So far, 10 different human TLRs have been recognized, with different subcellular distributions, and play crucial roles in the human innate immune response via inflammatory cytokines [8, 10]. TLR1, TLR2, TLR4, TLR5, TLR6, and TLR10 are present on the cell surface and can identify membrane lipids of microbes. However, the endosomal receptors TLR3, TLR7, TLR8, and TLR9 have the ability to identify host-derived nucleotides and microbial pathogens and trigger inflammatory responses [11]. TLR1 and TLR6 form heterodimers with TLR2, which are crucial in mucosal immune response regulation [12]. The mRNA expression of TLRs is found in many different types of cancers and plays a fundamental role in cancer occurrence and progression through the regulation of cell survival and proliferation. For instance, it was found that TLR1, TLR6, and TLR7 were upregulated in the human non-small-cell lung cancer cell line SPC-A1 [13]. Furthermore, the *TLR2* and *TLR6* genes were highly expressed in human melanoma cells [14] and in patients with ovarian cancer[14]. The clinical significance of the expression levels of TLR1, TLR2, TLR4, TLR5, TLR6, TLR8, and TLR10 in human renal carcinoma cells, relative to the levels in normal renal cells, has been reported [15].

Notably, genetic factors have been observed to be principal causes of BC development[16]. Genetic variations of single nucleotide polymorphisms (SNPs) normally occur in the human genome and have been comprehensively examined in genetic cancer studies[17]. Recent studies have revealed that SNPs of multiple genes are associated with the initiation of tumorigenesis, including BC risk [17, 18]. In 2015, Cotterchio and colleagues revealed that polymorphism in *TLR6* was associated with the risk of pancreatic cancer [19]. Additionally, *TLR1* and *TLR6* are located within the same gene cluster [20] and polymorphisms in these genes have been reported to increase advanced prostate cancer risks [21]. Previous studies have shown that a variant of *TLR6* rs13281615 was associated with increased BC risk in the Chinese population [22]. The *TLR6* SNPs rs3796508 and rs5743810 have been reported in different types of diseases, but not in any malignancies. Therefore, the current study aimed to identify the potential association between *TLR6* rs3796508 and rs5743810 and increased BC risk by comparing their frequencies in Saudi Arabian women with BC and in healthy women. Additionally, the potential use of these SNPs as diagnostic biomarkers for BC was evaluated.

## Results

### Basic clinical characteristics of the study subjects

All of the study participants were of Saudi Arabian ethnicity and exclusively female. The selected clinical characteristics of the included population are shown in Table 1. There were no variations in either demographic or other pertinent characteristics between the BC cases and healthy controls; however, there were some clinical differences for other aspects. The BC group consisted of 127 women (median age at diagnosis 48 ± 8.2 years), who were selected from the Oncology Centre at King Faisal Specialist Hospital & Research Center from 2002 to 2004. No significant differences in age and gender distribution were seen between the BC group and the control group. The control group was age-matched to the BC group (i.e., median age 48 years) and comprised 116 healthy women who had neither a family nor a personal history of BC. Of the 127 cases in the BC group, 45 were aged ≤48 years and 82 were aged >48 years. By contrast, in the control group, 62 women were ≤48 years of age and 54 were >48 years of age. In addition, the family histories of BC and health-affecting behaviors (e.g., smoking habit) were found to be similar between the two study groups (Table 1). Other clinical data for those populations, including stage of BC, medications, presence of other diseases, and status of the estrogen receptor (ER^+^ and ER^–^), human epidermal growth factor receptor (positive and negative), and progesterone receptor (positive and negative), were collected and are summarized in Table 1.

**Table 1:** Study population characteristics among Saudi BC cases and healthy controls used for genotyping.

Variable	Parameter	Cases N (%)	Controls N (%)
**Total persons**	-	**127**	**116**
**Age (Years): Median age (48±8.2)**	≤48	45 (35.43%)	62 (53.49)
	>48	82 (64.57%)	54 (46.55%)
**Estrogen receptor**	ER+	76 (59.84%)	NA
	ER-	49 (38.58%)	NA
**Progesterone receptor**	PR+	71 (55.91%)	NA
	PR-	55 (43.31%)	NA
**HER Status**	HER+	49 (38.59%)	NA
	HER-	78 (61.42%)	NA
**Smoking habits**	Smokers	0 (0%)	0(0%)
	No Smokers	127 (100%)	116 (100%)

Abbreviations: BC, breast cancer; NA, not applicable.

### Association of *TLR6* gene polymorphisms with BC risk in Saudi Arabian female patients

In the current study, the distributions of the two *TLR6* SNPs (rs3796508 G/A and rs5743810 C/T) did not deviate significantly from HWE (*P* > 0.05). The genotypic and allelic distributions of the two *TLR6* SNPs among the BC patients and healthy controls are presented in Table 2. Association analysis revealed no significant differences in genotypic and allelic frequencies between the BC group and control group for *TLR6* rs3796508 (Table 3). However, the genotypic frequencies of rs3796508 G/A in the BC group were 94%, 6%, and 0% for Val/Val, Val/Met, and Met/Met, respectively, whereas they were 93%, 6%, and 1%, respectively, in the control group. The homozygous “Met/Met” genotype of rs3796508 did not indicate any association with BC cases when compared with the healthy controls (OR = 0.291, CI = 0.012–7.216, *P* = 0.285; see Table 3). For rs5743810 C/T, the Ser/Pro genotype was present in 28% of the BC patients and in 3% of the control subjects. Interestingly, there were significant differences in the frequency of the Pro allele of this SNP between the two groups, with 86% of BC patients and 98% of healthy controls carrying the allele (OR = 0.103, CI = 0.036–0.293, *P* = 0.00001; Table 3).

**Table 2:** Distribution of genotypes and allele frequencies of the TLR6 gene loci among Saudi BC cases and controls.

Genotype	Cases	HWE P-value	Controls	HWE P-value
**rs3796508**				
**Val/Val**	117	0.047396*	102	0.852705
**Val/Met**	8		7	
**Met/Met**	0		1	
**rs5743810**				
**Ser/Ser**	0	0.711682	0	0.062743
**Ser/Pro**	36		4	
**Pro/Pro**	91		116	

**Table 3:** Genotype and allele frequencies of TLR6 gene polymorphism in Saudi BC cases and controls.

SNP ID	Genotype	Cases	Controls	OR	(95% CI)	^χ2^ –Value	P*- Value
		**n = 125**	**n = 110**				
	**Val/Val**	**117** (0.94)	**102** (0.93)	Ref			
**rs3796508**	**Val/Met**	**8** (0.06)	**7** (0.06)	0.996	0.349–2.843	0.0009	0.99453
	**Met/Met**	**0** (0.00)	**1** (0.01)	0.291	0.012–7.216	1.14	0.28542
	**Val/Met +Met/Met**	**8** (0.06)	**8** (0.07)	0.872	0.316–2.406	0.07	0.79099
	**Val**	**242** (0.97)	**211** (0.96)	Ref			
	**Met**	**8** (0.03)	**9** (0.04)	0.775	0.294–2.045	0.27	0.60574
		**n = 127**	**n = 120**				
	**Ser/Ser**	**0** (0.00)	**0** (0.00)	Ref			
**rs5743810**	**Ser/Pro**	**36** (0.28)	**4** (0.03)	8.111	0.14–461.1	—	1
	**Pro/Pro**	**91** (0.72)	**116** (0.97)	0.785	0.015–39.962	—	1
	**Ser/Pro +Pro/Pro**	**127** (1)	**120** (1)	1.058	0.021–53.747	—	1
	**Ser**	**36** (0.14)	**4** (0.02)	Ref			
	**Pro**	**218** (0.86)	**236** (0.98)	0.103	0.036–0.293	25.94	<0.00001*

* p <0.05

### Correlation of the genotypic distribution of *TLR6* gene polymorphisms with clinical parameters of the two study groups

We further evaluated the *TLR6* genotypes in relation to the different demographic and clinical variables. Advanced analysis of the genotypic distribution of the SNPs based on the age at BC diagnosis revealed that the frequency of rs3796508 distribution did not appear to be associated with the risk of BC development in both the younger (≤48 years) and older (>48 years) populations. However, the distributions of rs3796508 genotypes in BC patients ≤48 years of age were 91%, 9%, and 0% for Val/Val, Val/Met, and Met/Met, respectively, whereas those in the control patients were 98%, 2%, and 0%, respectively. In addition, the frequencies of the heterozygous “Val/Met” genotype of rs3796508 were similar between the BC and control groups in both age-based subpopulations (i.e., ≤48 years and >48 years) (Table 4). By contrast, rs5743810 showed a significant association with protection against advanced BC risk in the patients who were diagnosed with BC at both ≤48 and >48 years of age. However, based on the allelic frequencies, the Pro allele gave a 9-fold higher protection against the risk of BC than did the Ser allele in Saudi Arabian women in both age-based subpopulations (OR = 0.101, CI = 0.022–0.455, *P* = 0.00037 for women aged ≤48 years; and OR = 0.120, CI = 0.028–0.519, *P* = 0.00087 for women aged >48 years) (Table 4).

**Table 4:** Genotype and allele frequencies of TLR6 SNPs in Saudi BC cases versus controls based on age of diagnostic.

SNP ID	Genotype	Cases	Controls	OR	(95% CI)	χ^2^ Value	P*- Value
**patient’s age ≤ 48 years**							
**rs3796508**		**n = 44**	**n = 56**				
	Val/Val	**40**(0.91)	**55**(0.98)	Ref			
	Val/Met	**4**(0.09)	**1**(0.02)	5.500	0.592–51.090	2.77	0.09615
	Met/Met	**0**(0.00)	**0**(0.00)	1.370	0.027–70.519	—	1
	Val/Met +Met/Met	**4**(0.09)	**1**(0.02)	5.500	0.592–51.090	2.77	09615
	Val	**84**(0.95)	**111**(0.99)	Ref			
	Met	**4**(0.05)	**1**(0.01)	5.286	0.580–48.162	2.70	0.18404
**rs5743810**		**n = 45**	**n = 55**				
	Ser/Ser	**0**(0.00)	**0**(0.00)	Ref			
	Ser/Pro	**14**(0.31)	**2**(0.04)	5.800	0.092–365.479	—	1
	Pro/Pro	**31**(0.69)	**53**(0.96)	0.589	0.011–30.413	—	1
	Ser/Pro+Pro/Pro	**45**(1)	**55**(1)	0.820	0.016–42.132	—	1
	Ser	**14**(0.16)	**2**(0.02)	Ref			
	Pro	**76**(0.84)	**108**(0.98)	0.101	0.022–0.455	12.69	0.00037*
**SNP ID**	**Genotype**	**Cases**	**Controls**	**OR**	**(95% CI)**	^**χ2**^ **Value**	P*- Value
**patient’s age > 48 years**							
**rs3796508**		**n = 81**	**n = 54**				
	Val/Val	**77** (0.95)	**47** (0.87)	Ref			
	Val/Met	**4** (0.05)	**6** (0.11)	0.407	0.109–1.517	1.89	0.16922
	Met/Met	**0** (0.00)	**1** (0.02)	0.204	0.008–5.118	1.62	0.20350
	Val/Met +Met/Met	**4** (0.05)	**7** (0.13)	0.349	0.097–1.256	2.79	0.09499
	Val	**158** (0.98)	**100** (0.93)	Ref			
	Met	**4** (0.02)	**8** (0.07)	0.316	0.093–1.078	3.72	0.07084
**rs5743810**		**n = 82**	**n = 55**				
	Ser/Ser	**0** (0.00)	**0** (0.00)	Ref			
	Ser/Pro	**22** (0.27)	**2** (0.04)	9.000	0.144–560.703	—	1
	Pro/Pro	**60** (0.73)	**53** (0.96)	1.131	0.022–57.981	—	1
	Ser/Pro+Pro/Pro	**82** (1)	**55** (1)	1.486	0.029–76.027	—	1
	Ser	**22** (0.13)	**2** (0.02)	Ref			
	Pro	**142** (0.87)	**108** (0.98)	0.120	0.028–0.519	11.08	0.00087*

* = P<0.05, Ref = Reference allele

Finally, we analyzed the associations of the genotypic and allelic frequency distributions of rs3796508 and rs5743810 with the ER status of the BC patients. In the case of rs3796508, no significant associations were found with either ER^+^ or ER^−^status compared with the controls (Table 5), as the genotypic frequencies of this SNP were similar between both groups analyzed. In the BC group, 95%, 5%, and 0% carried the Val/Val, Val/Met, and Met/Met genotypes, respectively, whereas the distributions were 93%, 6%, and 1%, respectively, for the control group. However, based on the ER^+^ or ER^−^status, the rs5743810 Pro allele frequency was again more greatly associated with BC risk protection in this ethnic group than was the Ser allele (OR = 0.100, CI = 0.034–0.297, *P* = 0.00001 for ER^+^ BC cases; and OR = 0.102, CI = 0.033–0.318, *P* = 0.00001 for ER^−^BC cases) (Table 5). Moreover, the distribution frequencies of genotypes Ser/Ser, Ser/Pro, and Pro/Pro were similar in the ER^+^/ER^−^subgroups of BC patients compared with the control group (Table 5).

**Table 5:** Genotype and allele frequencies TLR6 SNPs in Saudi BC cases and controls based on ER+/ER^-^ status.

**SNP ID**	**Genotype**	**Cases**	**Controls**	**OR**	**(95% CI)**	^**χ2**^ **Value**	P*- Value
**ER+**							
**rs3796508**		**n = 75**	**n = 110**				
	Val/Val	**71** (0.95)	**102** (0.93)	Ref			
	Val/Met	**4** (0.05)	**7** (0.06)	0.821	0.232–2.909	0.09	0.75954
	Met/Met	**0** (0.00)	**1** (0.01)	0.478	0.019–11.898	0.69	0.40504
	Val/Met +Met/Met	**4** (0.05)	**8** (0.07)	0.718	0.208–2.477	0.28	0.59899
	Val	**146** (0.97)	**211** (0.96)	Ref			
	Met	**4** (0.03)	**9** (0.04)	0.642	0.194–2.125	0.53	0.46507
**rs5743810**		**n = 76**	**n = 120**				
	Ser/Ser	**0** (0.00)	**0** (0.00)	Ref			
	Ser/Pro	**22** (0.29)	**4** (0.03)	5.000	0.087–286.553	—	1
	Pro/Pro	**54** (0.71)	**116** (0.97)	0.468	0.009–23.889	—	1
	Ser/Pro+Pro/Pro	**76** (1)	**120** (1)	0.635	0.012–32.331	—	1
	Ser	**22** (0.14)	**4** (0.02)	Ref			
	Pro	**130** (0.86)	**236** (0.98)	0.100	0.034–0.297	24.65	<0.00001*
**SNP ID**	**Genotype**	**Cases**	**Controls**	**OR**	**(95% CI)**	^**χ2**^ **Value**	P*- Value
**ER-**							
**rs3796508**		**n = 48**	**n = 110**				
	Val/Val	**44** (0.92)	**102** (0.93)	Ref			
	Val/Met	**4** (0.08)	**7** (0.06)	1.325	0.369–4.756	0.19	0.66555
	Met/Met	**0** (0.00)	**1** (0.01)	0.768	0.031–19.214	0.43	0.51193
	Val/Met +Met/Met	**4** (0.08)	**8** (0.07)	1.159	0.332–4.051	0.05	0.81698
	Val	**92** (0.96)	**211** (0.96)	Ref			
	Met	**4** (0.04)	**9** (0.04)	1.019	0.306–3.394	0.0009	1
**rs5743810**		**n = 49**	**n = 120**				
	Ser/Ser	**0** (0.00)	**0** (0.00)	Ref			
	Ser/Pro	**14** (0.29)	**4** (0.03)	3.222	0.056–186.825	—	1
	Pro/Pro	**35** (0.71)	**116** (0.97)	0.305	0.006–15.635	—	1
	Ser/Pro+Pro/Pro	**49** (1)	**120** (1)	0.411	0.008–20.993	—	1
	Ser	**14** (0.14)	**4** (0.02)	Ref			
	Pro	**84** (0.86)	**236** (0.98)	0.102	0.033–0.318	21.98	<0.00001*

* = P<0.05, Ref = Reference allele

### In silico analysis

Different bioinformatics algorithms and software were used to predict the pathogenicity and damaging effects of the rs5743810 (Ser249Pro) and rs3796508 (Val327Met) mutations. The majority of the algorithms considered these two SNPs as being neutral, with harmless effects, and there was no evidence of deleterious events correlated with the mutations. This exclusion of negative effects of the mutations by the in silico analysis supported our findings that these SNPs might have some positive effects (Table 6). Proline is considered to be a unique amino acid with a specific structure, where the side chain is connected to the protein backbone twice, creating a 5-membered nitrogen ring. Therefore, any mutation that introduces a Pro residue at a site where the backbone torsion angles are unable to accommodate such a residue is usually associated with clashes and damaging effects because of the energy changes. Nevertheless, our in silico predictions indicated that there were no damaging effects associated with rs5743810 (Ser249Pro) and s3796508 (Val327Met), confirming that these two SNPs are protective mutations.

**Table 6:** Predict the pathogenicity and damaging effects of the rs5743810 (Ser249Pro) and rs3796508 (Val327Met) mutations.

Mutation nucleotide	Protein Change	Mutation taster	Polyphen-2.0	SIFT	PROVEAN	Mutation Assessor	FATHMM	CONDEL
c.C>T	p.V327M	(0.99) Harmless polymorphism	(0.069) Neutral	(0.02) Damaging	(-0.716) Neutral	(2.015) Predicted functional (low-Medium)	(3.03) Tolerated	(0.362) Neutral
c.A>G	p.S249PRO	(0.99) Harmless polymorphism	(0.000) Neutral	(0.56) Neutral	(1.124) Neutral	(-1.06) Predicted non-functional (Neutral)	(2.49) Tolerated	(0.326) Neutral

**Fig 1:**
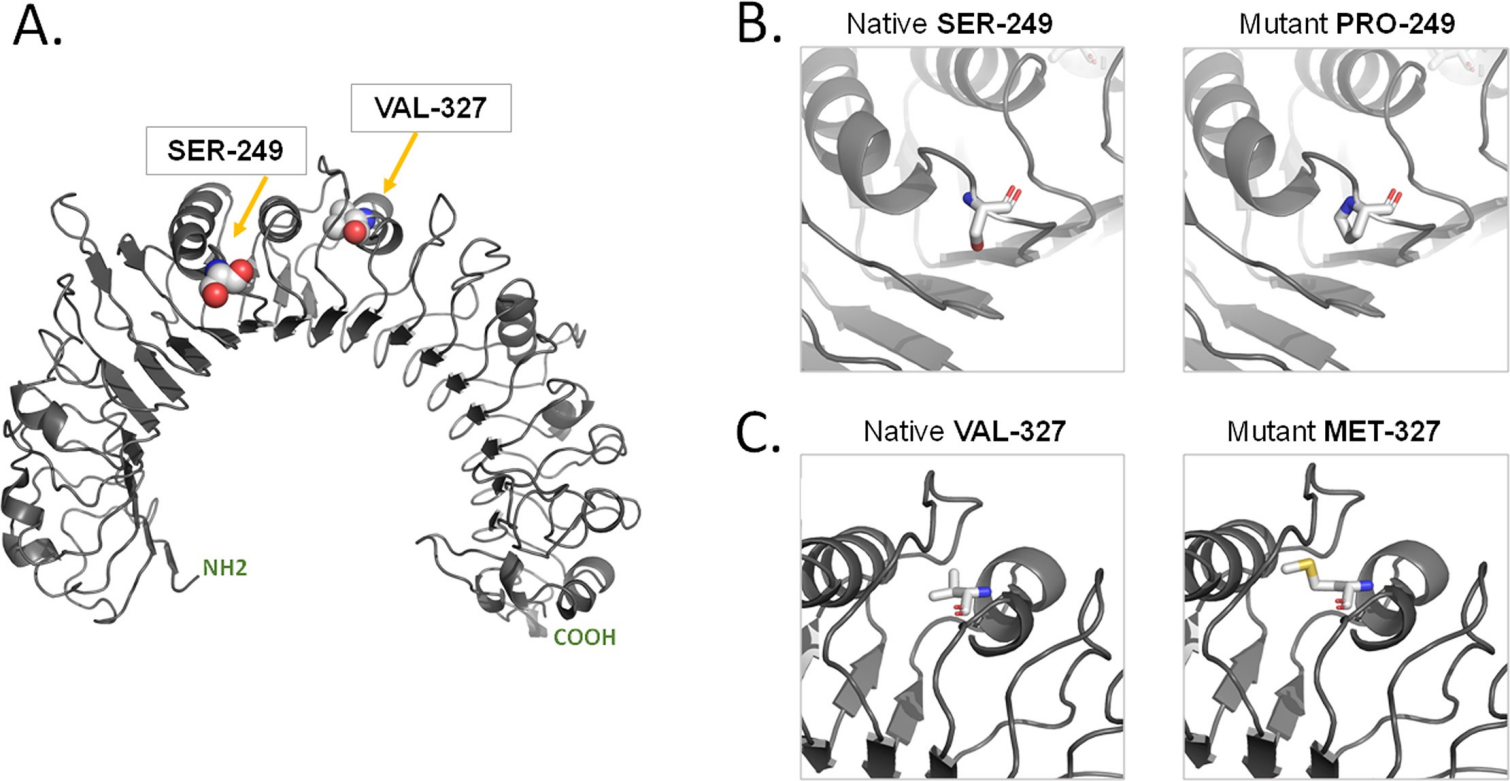
**Structural function of TLR6:** A) Homology model, showing the location of Ser-249 and Val-327 in spheres representation. B) Native Ser-249 and Mutant Pro-249 in sticks representation. C) Native Val-327 C. Mutant Met-327 in sticks representation. CPK Colours used for illustration.

On the basis of the solved crystal structure of the Toll/interleukin-1 receptor domain of TLR6 (PDB 4OM7) from positions 640 to 782, a homology model was constructed using the SWISS-MODEL template library (SMTL version 2017-10-23, PDB release 2017-10-13), which models tertiary and quaternary protein structures using evolutionary information. The best template structure matching the target sequence was selected (3a79.1.B), having 66% sequence identity and a resolution of 2.90 Angstrom. The model revealed that Glu33-Lys573 has an overall curved shape. Using the “mutation” tool in SWISS-MODEL, Ser249 was mutated to Pro249, and Val327 was mutated to Met327 (Fig 1). The original side chain was substituted with the best rotamer with the lowest energy score of the Thr50 amino acid.

## Discussion

Polymorphisms in different genes are known to be caused by genetic changes in the DNA composition [23]. SNPs may result in DNA genetic variations, which may change the gene-encoded protein function and/or activity by means of amino acid exchange [24]. We hypothesized that the *TLR6* gene could play a vital role in innate immune system activation, and that dysregulation of this gene function may result in the development of metastatic BC. It was previously reported that genetic changes in TLRs genes are correlated with increased BC risk [3, 25] and several TLR gene polymorphisms have been identified to be related to increased BC susceptibility [26]. The primary objective of the present study was to investigate the association of *TLR6*-associated SNPs with increased risk of BC development in Saudi Arabian women. Specifically, the SNPs studied were rs3796508 (979 G>A) and rs5743810 (745 C>T), located on exonic regions of chromosome 4, with valine changed to methionine for rs3796508 (Val327Met) and serine changed to proline (Ser249Pro) for rs5743810. Assessment of rs3796508 revealed no association between its genotypic and allelic frequency distributions with risk of BC in this population. So far, no previous study has reported a clear association between this polymorphism and the development of cancer, including BC. Thus, the rs3796508 polymorphism can affect TLR6 expression in the women suffering from BC without being the main cause of the disease development. However, the significantly higher frequency of the minor Pro allele of rs5743810 indicated that it had a strong association with protection against both BC development and metastatic risks in Saudi Arabian women, both in the youngest and the oldest cases and also in the different types of BC (ER^+^ or ER^–^). The rs5743810 SNP is described in the literature as a downstream gene variant, which means that it is located in an exonic region of the gene. This localization means that rs5743810 may have an effect on TLR6 expression and function. The change in amino acid from Ser to Pro results in functional deterioration of TLR6 and predisposes individuals to dysregulation of the innate immune system, which can potentially cause development of the cancer. It is believed that polymorphisms in coding regions are currently relevant to cancer development owing to their roles in gene expression regulation. TLR6 functionally interacts with TLR2 to mediate the cellular response to bacterial lipoproteins and activate the NF-κB pathway and inflammatory events, and consequently, may contribute to tumor development and progression [27–29]. Despite the enhanced expression and activation of TLR6, its function and role in cancer are not clearly understood. Upon TLR2/TLR6 stimulation, the Pro phenotype of the *TLR6* rs5743810 SNP was found to be associated with lower secretions of proinflammatory cytokines as well as tumor necrosis factor-alpha and IL-6 (among others), decreasing the risk of BC development, as compared with the Ser or wild-type phenotype responses. A Ser249Pro polymorphism in the extracellular domain of the encoded protein may be associated with BC susceptibility in some populations. Some studies on clinical samples have pointed to TLR6-related tumor progression. Rogers et al. 2013 [30] observed an association between sequence variants in *TLR6* and prostate cancer susceptibility among men of African descent[30]. Furthermore, TLR6 expression was also associated with smoking [31, 32].

Our Structure model of TLR-6 illustrate the importance of the in silico evaluation of functional significance and representing how the Ser/Pro change affect ligand induced dimerization. Briefly, Proline is considered to be a unique amino acid with a specific structure, where the side chain is connected to the protein backbone twice, creating a 5-membered nitrogen ring. Therefore, any mutation that introduces a Pro residue at a site where the backbone torsion angles are unable to accommodate such a residue is usually associated with clashes and damaging effects because of the energy changes. Ser/Pro change is near the dimerization interface however this dimerization is not directly interfering with Ser/Pro change. Ser/Pro change is also towards the end of an alpha-helical LRR segment: possibly this could alter the conformation of LRR9 which is involved in ligand binding [Ref 1.]. Nevertheless, our in silico predictions indicated that there were no damaging effects associated with rs5743810 (Ser249Pro) and s3796508 (Val327Met), confirming that these two SNPs are protective mutations. These outcomes may show that the rs5743810 (Ser249Pro) SNP of the *TLR6* gene may be distributed differently among diverse cancer types and ethnicities, and can thus be used as a biomarker for the diagnosis of different cancers (rather than a specific marker for BC). However, no data on the functional implications of this SNP on TLR6 expression are currently available.

## Conclusions

### Novelty and impact

Breast cancer (BC) remains the most prevalent cancer among female worldwide. The lethal disease represents 19.9% of the total cancer-related deaths in Saudi Arabia women and ranked as the first leading cause of deaths. Due to the high rate of the consanguineous marriage in SA, several genetics basis diseases become more prevalence, including BC. Therefore, studying the association of the *TLR6* SNPs with BC susceptibility in Saudi Arabian women is highly needed.

The present study clarifies significant roles for *TLR6* polymorphic variants in determining BC susceptibility risk among the Saudi Arabian female population, especially that of rs5743810. Additionally, the in silico analysis indicates a protective role for the rs5743810 mutation. However, further investigations are necessary to address the association owing to the limited number of BC samples in the present study. In addition, further independent studies of genetic correlations between *TLR6* sequence variants and BC risk in other ethnic populations would be of great interest. Because the exact mechanisms by which *TLR6* polymorphisms contribute to cancer pathogenesis are unknown, such elucidation will be a challenge for future research. We believe that these SNPs and their BC risk associations could contribute to their development as biomarkers for the early detection of BC.

## Materials and methods

### Ethical approval certificate

This case-controlled study was accredited by the Ethics Review Committee at King Faisal Medical City (Ethical approval number 15-089E), and the panel members assented to the use of a consent form. Prior to sample collection, all participating study subjects completed a questionnaire for demographic information, such as the patient’s age at diagnosis, gender, family health history, past medical history, tumor location, tumor characteristics, and reproductive records. The estrogen receptor level was determined via immunohistochemical analysis. All participants in the current investigation agreed to publication of the data generated from this study.

### Study design and patient recruitment

The present case-controlled BC study included 127 Saudi Arabian women with histologically confirmed BC between 2011 and 2016 at King Faisal Medical City (Riyadh, Kingdom of Saudi Arabia) prior to any medical treatment. Whole blood specimens were obtained from the BC patients for genotyping were from doctor Abdulrahman Al Naeem at Department of Women's Imaging, King Fahad Medical City, Riyadh, Saudi Arabia. Additionally, blood specimens were collected from 116 healthy women who lacked any clinical signs of BC were from doctor Sana Abdulla Ajaj at Family Medicine Department, College of Medicine, King Saud University, Riyadh, Saudi Arabia. All subjects provided informed written consent to participate in the study and to approve the use of their biological samples for genetic analyses. The design of the study was approved by the local Ethics Committee number 15-089E from King Faisal Medical City, Riyadh and our study don't include any minorsGenomic DNA was extracted from all blood specimens. Both the BC patients and the healthy controls were frequency matched within five years by age and sex. Healthy female volunteers were allowed to participate in the study if they had a normal mammography and no previous cancer history.

### DNA extraction

For both groups of women, 3 mL of blood was drawn into EDTA-vacutainer vials (BD Vacutainer Systems, Plymouth, UK). Subsequently, genomic DNA was immediately isolated from 200 μL of the EDTA-anticoagulated blood using a QIAmp DNA Blood Mini Kit (Qiagen, Valencia, CA, USA) in accordance with the manufacturer’s instructions. The DNA samples were preserved at –20°C until used. The isolated genomic DNA concentration was measured with a Nano-Drop 8000 spectrophotometer (Thermo Scientific, Waltham, MA, USA), and the DNA purity was evaluated by measuring the absorbance ratios A_260_/A_280_ and A_260_/A_230_.

### Selected *TLR6* polymorphisms and DNA genotyping

Two SNPs in the *TLR6* gene were investigated and genotyped using the TaqMan allelic discrimination assay. Three selection criteria for the *TLR6* gene polymorphisms were used: (1) their positions, (2) their frequencies and their affiliations with cancer peril, as mentioned formerly, and (3) their correlations with many cancers in various ethnic groups, as reported in various literature reviews. The *TLR6* polymorphisms used in the current study—rs3796508 (979 G>A, Val327Met) and rs5743810 (745 C>T, Ser249Pro)—are in the exonic region. It is currently believed that polymorphisms in coding regions are relevant to cancer development owing to their roles in gene expression regulation (S1 Table). Sample genotyping was executed in a 96-well format by applying a QuantStudio 7 Flex Real-Time PCR system (Applied Biosystems, Foster City, CA, USA). In brief, each genotyping reaction was performed in an overall mixture volume of 10 μL containing DNA at a final amount of 15 ng, 5.6 μL of 2× TaqMan Genotyping Master Mix (Applied Biosystems), and 0.2 μL of 40× TaqMan Genotyping SNP Assay reagent (Applied Biosystems).

All primer sequences and probe mixtures were purchased from Applied Biosystems. The efficiencies of the marker and sample genotyping were validated through the performance of both positive and negative controls to confirm the genotyping quality. Genotyping of >10% of randomly selected samples was duplicated to obtain 100% identical results.

### In silico analysis

To determine the structural functions along with the effects of the rs5743810 (Ser249Pro) and rs3796508 (Val327Met) mutations, several in silico algorithmic software were used; namely, MutationTaster, PolyPhen-2.0 [33], SIFT[34], PROVEAN [35], MutationAssessor [36], FATHMM,[37] and CONDEL[38]. A homology model of the 3D structure of TLR6 was built using the SWISS-MODEL template library (SMTL version 2017-10-23, PDB release 2017-10-13) [39].

### Statistical analysis

Statistical analysis of the data was conducted using the SPSS version 16.0 statistical package (SPSS, Chicago, IL, USA). All probability values (*P* values) below 0.05 were considered statistically significant. Differences in the allelic and genotypic association of each SNP were tested for Hardy-Weinberg equilibrium (HWE). The odds ratios (ORs) and 95% confidence intervals (CIs) were calculated using the Chi-squared test to compare genetic variations between the BC patients and the healthy participants.

## Supporting information

S1 Table Characteristics of the TLR6 SNPs. TLR6_HUMAN : NM_006068 NP_006059 p.Val327Met
TLR6_HUMAN : NM_006068 NP_006059 p.Ser249Pro.
(DOCX)

## Abbreviations

BC: breast cancer
CI: confidence interval
ER: estrogen receptor
OR: odds ratio
SNPs: single nucleotide polymorphisms
TLR6: Toll-like receptor 6
TLRs: Toll-like receptors
HWE: Hardy-Weinberg equilibrium (HWE)
Pro: Proline
Ser: Serine
Val: Valine
Met: Methionine
