# Supplementary material for: Novel sequence variants in the TLR6 gene associated with advanced breast cancer risk in the Saudi Arabian population
Source: PLoS One. 2018 Nov 2;13(11):e0203376. doi: 10.1371/journal.pone.0203376 (PMC6214682; doi:10.1371/journal.pone.0203376)
Supplement: S1 Table — TLR6_HUMAN : NM_006068 NP_006059 p.Val327Met TLR6_HUMAN : NM_006068 NP_006059 p.Ser249Pro. (DOCX) [file pone.0203376.s001.docx]

**Table S1**: Characteristics of the TLR6 SNPs

TLR6_HUMAN : NM_006068 NP_006059 p.Val327Met

TLR6_HUMAN : NM_006068 NP_006059 p.Ser249Pro

| CHR | STRAND | START | | REF | | ALT | | TRANSCRIPT | | PROTEIN | | dbSNP | AA_POS | | | AA_REF | AA_ALT | | | UniProt_ID | SWISSPROT_ID | |  |
| --- | --- | --- | --- | --- | --- | --- | --- | --- | --- | --- | --- | --- | --- | --- | --- | --- | --- | --- | --- | --- | --- | --- | --- |
| 4 | - | | 38830116 | | C | | T | | ENST00000381950 | | ENSP00000371376 | rs3796508 | | 327 | V | | | M | Q9Y2C9 | | | TLR6_HUMAN | |
| 4 | - | | 38830350 | | A | | G | | ENST00000381950 | | ENSP00000371376 | rs5743810 | | 249 | S | | | P | Q9Y2C9 | | | TLR6_HUMAN | |
